# Hernie interne du ligament falciforme: une cause rare d'occlusion intestinale

**DOI:** 10.11604/pamj.2019.32.48.17845

**Published:** 2019-01-28

**Authors:** Hamza Hasnaoui, Ouadii Mouaqit, El Bachir Benjelloun, Abdelmalek ousadden, Khalid Ait Taleb, Hicham El Bouhaddouti

**Affiliations:** 1 Service de Chirurgie Viscérale A, CHU Hassan II, Fès, Maroc; 2 Faculté de Médecine et de Pharmacie, Université Sidi Mohamed Ben Abdellah de Fès, Maroc

**Keywords:** Hernie interne, ligament falciforme, occlusion intestinale, Hypochondrium, internal hernias, falciform ligament, Abdominal

## Abstract

Les hernies internes sont une cause rare d'occlusion intestinale aiguë. La hernie à travers le ligament falciforme est une forme exceptionnelle dont le diagnostic est souvent fait en peropératoire. La tomodensitométrie abdominale, pratiquée en urgence, peut aider au diagnostic en préopératoire et permet de guider l'attitude thérapeutique. Aussi, nous a-t-il paru opportun de rapporter ce cas colligé dans le service de chirurgie viscérale A du CHU Hassan II de Fès. Nous rapportons l'observation d'un patient âgé de 48 ans, sans antécédent particulier, admis aux urgences avec un tableau d'occlusion évoluant depuis 4 jours. La radiographie d'abdomen sans préparation objectivait de multiples niveaux hydro-aériques de type grêlique dont certains se projetaient en regard de l'air hépatique, ainsi que la présence d'une anse intestinale plate en continuité avec un segment intestinal distendu. La tomodensitométrie abdominale n'a pas été réalisée vu une fonction rénale qui était altérée. Le patient était alors opéré en urgence après mise en condition et le diagnostic d'une hernie interne du ligament falciforme était fait en peropératoire. La hernie interne à travers le ligament falciforme est une cause rare d'occlusion intestinale aiguë de l'adulte dans notre pratique quotidienne. Le diagnostic est le plus souvent fait en peropératoire. Il faut savoir y penser devant le jeune âge, l'absence d'antécédents de chirurgie abdominale ou de processus infectieux intra péritonéal et la présence de niveaux hydro-aériques dans l'hypocondre droit.

## Abstract (en)

Internal hernias are a rare cause of acute intestinal obstruction. Hernias through the falciform ligament is uncommon. Diagnosis is often made intraoperatively. Abdominal CT scan, performed in an emergency situation, can enable preoperative diagnosis and guide the therapeutic approach. In this respect, we here report a case whose data were collected in the Department of Visceral Surgery at the University Hospital Hassan II in Fez. The study involved a 48-year old patient, with no particular previous history, admitted to the Emergency Department with occlusion evolving over 4 days. Abdominal x-ray without treatment objectified multiple hydroaeric levels in small bowel some of which projected towards the hepatic region as well as the presence of a flat intestinal loop in continuity with distended intestinal segment. Abdominal CT scan was not performed due to altered renal function. The patient then underwent emergency surgery after stabilization of his condition and the diagnosis of internal hernias through the falciform ligament was made intraoperatively. In adults, internal hernia through the falciform ligament is a rare cause of acute intestinal obstruction in our daily practice. The diagnosis is most often made intraoperatively. It is necessary to suspect it in young patients with no history of abdominal surgery or intraperitoneal infectious process and with hydroaeric levels in the right upper quadrant.

## Introduction

La hernie à travers le ligament falciforme est exceptionnelle et de diagnostic souvent peropératoire [1, 2]. Actuellement la tomodensitométrie abdominale pratiquée en urgence, peut aider au diagnostic en préopératoire et permet de guider l'attitude thérapeutique. A la lumière d'une observation et d'une revue de la littérature, le but de notre travail est de mettre en exergue les difficultés diagnostics et les différentes modalités thérapeutiques de cette pathologie.

## Patient et observation

**Figure 1:**
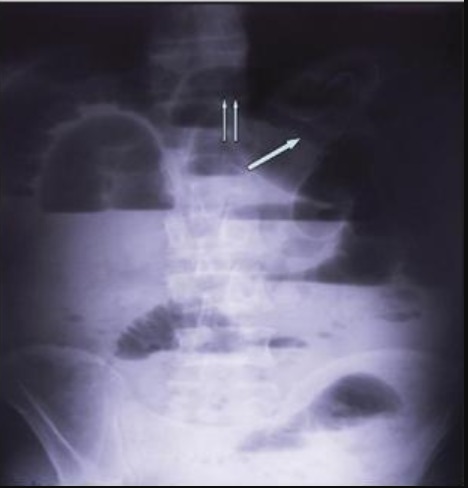
Radiographie d'abdomen sans préparation de face debout montrant de multiples niveaux hydro-aériques de type grêlique dont certains siègent au niveau de l'hypochondre droit. Présence d'une anse plate (flèche simple) contenant de l'air en continuité avec une anse distendue avec une zone de transition (flèche double) se projetant en regard de l'aire hépatique

**Figure 2:**
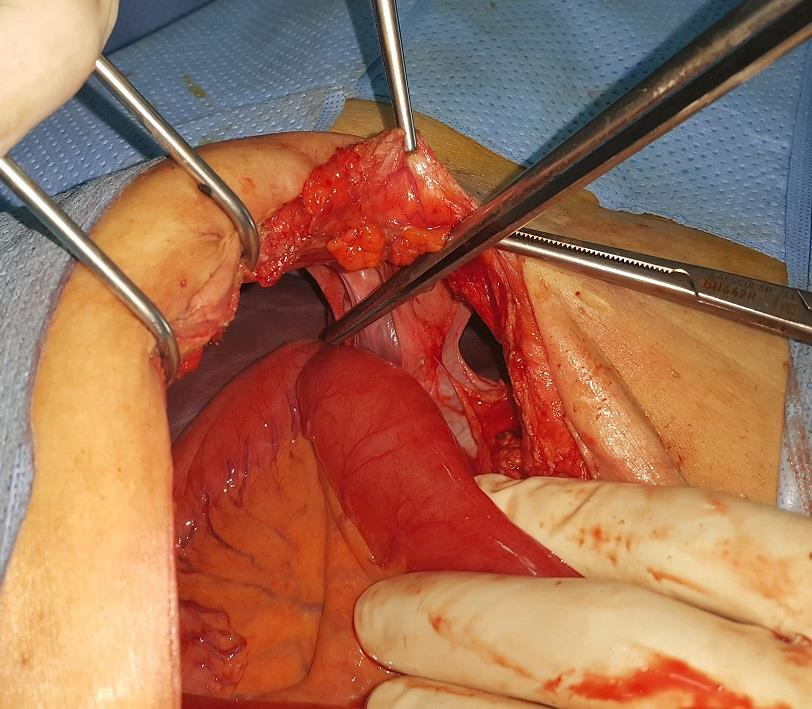
Vue opératoire montrant une hernie interne au niveau du ligament falciforme

**Figure 3:**
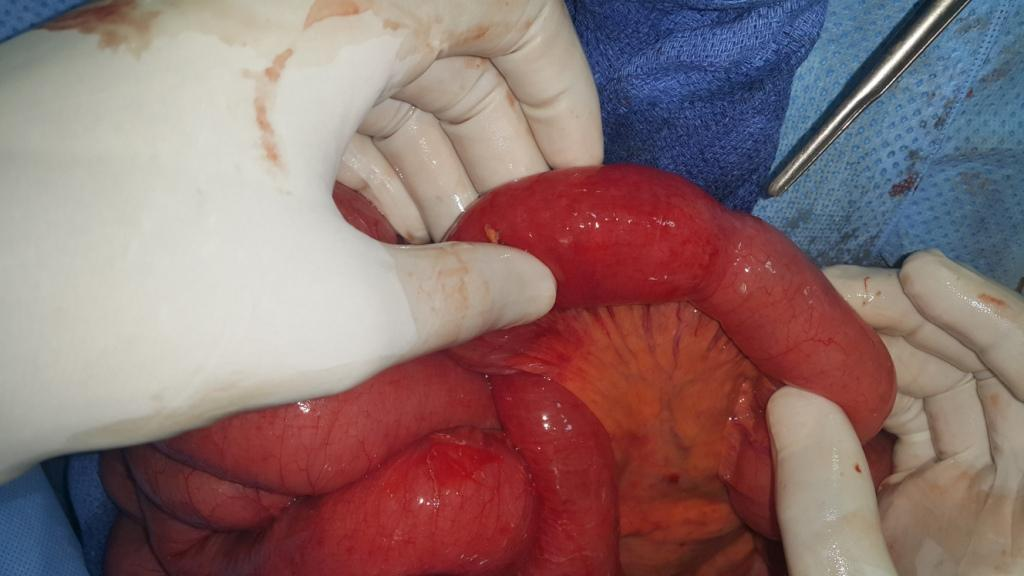
Vue opératoire après désincarcération des anses grêles

**Figure 4:**
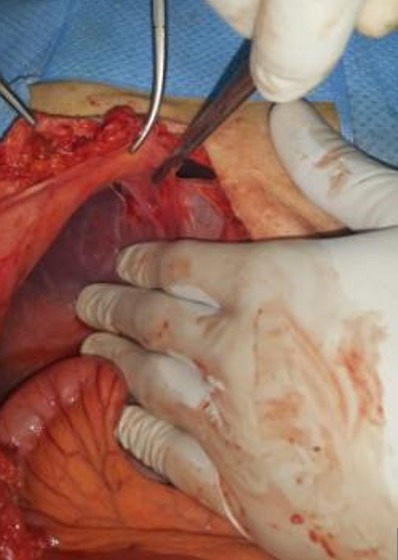
Vue opératoire durant l'effondrement du ligament falciforme de son insertion diaphragmatique

Un homme âgé de 48 ans, sans antécédents, admis aux urgences pour une symptomatologie évoluant depuis 4 jours faite de douleurs épigastriques à type de torsion, associées à des vomissements bilieux et un arrêt des matières et des gaz. L'examen physique trouvait un météorisme abdominal diffus avec une sensibilité abdominale plus marquée au niveau de l'épigastre. Les orifices herniaires étaient libres et le toucher rectal trouvait une ampoule vide. La radiographie de l'abdomen sans préparation objectivait de multiples niveaux hydro-aériques de type grêlique dont certains se projetaient en regard de l'air hépatique. Par ailleurs, on notait la présence d'une anse intestinale plate en continuité avec un segment intestinal distendu et dont la zone de transition se projetait au niveau de l'hypochondre droit (Figure 1). La tomodensitométrie n'a pas pu être faite suite à une insuffisance rénale fonctionnelle. L'hémogramme montrait une hyperleucocytose à 18 400 éléments/mm^3^, une protéine C réactive (CRP) à 61 mg/l, des troubles électrolytiques à l'ionogramme. Le diagnostic d'occlusion intestinale aiguë a été retenu. Mais devant l'aggravation de la sensibilité abdominale en défense, une laparotomie exploratrice était indiquée en urgence. L'exploration chirurgicale par une laparotomie médiane à cheval sur l'ombilic permettait de découvrir une hernie interne au niveau du ligament falciforme (Figure 2). L'intestin était de bonne vitalité et l'orifice herniaire faisait 3 cm de diamètre. Le geste opératoire avait consisté en une désincarcération du grêle (Figure 3) et un effondrement du ligament falciforme sur toute la longueur de son insertion diaphragmatique (Figure 4). Les suites opératoires étaient simples.

## Discussion

Les hernies internes sont des protrusions des viscères creux abdominaux dans un orifice intrapéritonéal, mais qui restent à l'intérieure de la cavité abdominale [3]. Elles constituent une cause rare d'occlusion intestinale aiguë chez l'adulte [4]. Selon leur siège, on distingue par ordre de fréquence décroissante les hernies para-duodénales, les hernies para-caecales, les hernies trans-mésentériques, les hernies méso-sigmoïdiennes et les hernies para-vésicales [3-6]. Les hernies à travers le ligament falciforme du foie sont exceptionnelles et représentent 0,1 à 0,3% de toutes les hernies internes [1-3]. Le diagnostic est rarement fait en préopératoire du fait de l'absence de spécificité des signes cliniques [6, 7]. Dans la majorité des cas, le diagnostic est fait à l'occasion d'un épisode hyperalgique aigu ou d'un syndrome occlusif comme dans notre observation. Le diagnostic doit être évoqué devant le jeune âge, l'absence d'antécédents de chirurgie abdominale ou de processus infectieux intrapéritonéal et la présence de niveaux hydro-aériques dans l'hypocondre droit [2]. La tomodensitométrie abdominale, non réalisée chez notre malade, est l'examen d'imagerie de référence devant un tableau d'occlusion intestinale aiguë sur un abdomen "vierge" [1, 6]. C'est un examen performant, permettant de faire un diagnostic précis de hernie interne dans 77% des cas, avec une sensibilité de 63% et une spécificité à 76% [8]. Dans le cadre d'une hernie interne à travers le ligament falciforme, la tomodensitométrie montre une convergence des plis et des vaisseaux mésentériques supérieurs vers la zone de striction constituée par le ligament falciforme [6, 8]. La tomodensitométrie permet également d'étudier le rehaussement de la paroi intestinale par le produit de contraste iodé, et ainsi de diagnostiquer une ischémie intestinale imposant une laparotomie dans les plus brefs délais. Le scanner abdominal ne doit en aucun cas retarder une intervention chirurgicale urgente en présence de signes cliniques ou biologiques de souffrance intestinale. Chez notre cas, en présence de signes cliniques et biologiques de gravité, la tomodensitométrie n'a pas pu être réalisée. L'orifice sur le ligament falciforme peut être d'origine congénitale, inflammatoire (satellite d'une cholécystite aigue) ou secondaire à une intervention chirurgicale (gastrectomie des 4/5) [2]. Chez notre malade l'absence d'antécédents opératoires et d'infection intrapéritonéale nous suggère une origine congénitale. Le viscère généralement hernié est l'intestin grêle mais le grand épiploon et le colon droit peuvent également être incarcéré [2, 5]. Une fois le diagnostic fait, la chirurgie en urgence s'impose pour réaliser une désincarcération de l'anse grêlique avec ou sans résection intestinale en fonction de sa vitalité. Nous avons traité le défect du ligament falciforme par l'effondrement de ce ligament sur toute la longueur de son insertion diaphragmatique afin de prévenir les récidives. Cette attitude chirurgicale a été également préconisée par Kobayashi *et al* [9]. La voie d'abord laparoscopique est faisable [9], elle offre plusieurs avantages par rapport à la voie classique, cependant, en cas de distension importante du grêle, elle risque d'être difficile et hasardeuse. Ceci explique notre choix pour la laparotomie dans notre observation.

## Conclusion

Les hernies internes sont une cause rare d'occlusion intestinale aigue. La hernie à travers le ligament falciforme est une forme exceptionnelle dont le diagnostic est souvent fait en peropératoire. La tomodensitométrie abdominale, pratiquée en urgence, peut aider au diagnostic en préopératoire et permet de guider l'attitude thérapeutique. Son traitement est chirurgical.

## Conflits d'intérêts

Les auteurs ne déclarent aucun conflit d'intérêts.
